# Short-term effects of irrigation and nitrogen management on paddy soil carbon pools under deep placement of basal fertilizer nitrogen

**DOI:** 10.1038/s41598-024-61931-5

**Published:** 2024-05-17

**Authors:** Jian Song, Jiaqi Wang, Quanying Hou, Zhenxiang Xing, Zhongxue Zhang, Sicheng Du, Mingyang Liu

**Affiliations:** 1 School of Water Conservancy and Civil Engineering, Northeast Agricultural University, Harbin, 150030 China; 2 Key Laboratory of Efficient Use of Agricultural Water Resources, Ministry of Agriculture and Rural Affairs of the People’s Republic of China, Northeast Agricultural University, Harbin, 150030 China

**Keywords:** Paddy, Irrigation regime, Nitrogen management, Active carbon fractions, Carbon pool management index, Ecology, Agroecology, Boreal ecology, Environmental sciences, Environmental impact

## Abstract

Active soil organic carbon (SOC) fractions are major driving factors of soil fertility. Understanding the effects of water and fertilizer management on changes in active SOC fractions helps improve soil quality and maintain high agricultural productivity. We conducted a 3-year field experiment in Northeast China. In this experiment, natural soil (CKT) was used as a blank, and two irrigation regimes were established: conventional flooded irrigation (FI) and controlled irrigation (CI). Four nitrogen application levels were set for both irrigation regimes under deep placement of basal fertilizer N: Nd0 (0 kg ha^–1^), Nd (110 kg ha^–1^), Nd1 (99 kg ha^–1^), and Nd2 (88 kg ha^–1^). After 3 years, at similar N fertilizer application rate, the rice yield, total organic carbon (TOC), and active SOC fraction content of CI were higher under CI than FI. The growth rate of rice yield was 3.8% − 8.63% under CI than FI. Under CI, the rice yield, active SOC fractions contents and carbon pool management index (CPMI) did not decrease with decreasing N application rate but instead reached the highest level in the CNd1 treatment. Overall, CI with Nd1 treatment appears to be the best practice for improving soil fertility and crop productivity in Northeast China.

## Introduction

Soil contains the largest terrestrial organic carbon (C) pool and is crucial in the global C cycle^1^. The content and quality of soil organic C (SOC) are important indicators that characterize the physical, chemical, and biological characteristics and processes of soil^2–4^. SOC provides the nutrients needed by crops, promotes water retention, improve soil physical properties, enhances the crop productivity^5^. Therefore, understanding SOC dynamics is important for the long-term sustainable development of agricultural ecosystems and the environment. However, total organic carbon (TOC) in soil becomes difficult to detect in the short term under a high C background. Soil easily oxidizable C (EOC), microbial biomass C (MBC), dissolved organic C (DOC), and other organic C fractions are sensitive indicators of soil quality, nutrient sources for biological growth, and energy sources for microorganisms and can reflect changes in the soil C pool caused by crop and environmental changes^6^. Therefore, these parameters serve as early indicators of changes in soil quality. The SOC content is usually in dynamic equilibrium, and agricultural management measures can affect this balance and thus affect SOC cycling. In addition, improper water management in paddy field can exacerbate greenhouse gas (GHG) emissions^7^, hence proper water management and precise management interventions are needed to sequester SOC and concomitantly decrease GHG emissions.

The C sequestration capacity of paddy soil is influenced by many factors, such as water management (irrigation regime) and fertilization (nitrogen fertilizer application rate)^8^. The management of water regimes is the most important measure in the paddy fields and impacts development, SOC, microbial composition, and other aspects^9^, thereby changing the distribution and trend of SOC^10^. For instance, Li et al. reported that irrigation methods significantly impact different SOC pools^11^. Xu^12^ reported that water-saving irrigation improves the stability of SOC in the 0–20 cm soil layer, increases the accumulation of SOC, and increases the soil C storage and C storage potential. Zhang et al.^13^ reported that higher soil carbon sequestration capacity and less carbon loss under CI than FI. Li et al.^14^ reported that compared with sufficient irrigation, insufficient irrigation has a higher cumulative effect on soil C than on C loss.

N fertilizer management is also an important part of agricultural field measures. The application of N fertilizer can affect the decomposition of SOC by impacting soil microbial activity, plant growth, root activity, litter decomposition, etc.^15^. Excessive application N fertilizer can exacerbate the GHG emissions and diffused pollution, leading to soil fertility decline^16–18^. Poeplau et al.^19^ found that the soil organic C fixation increased under N fertilizer applied treatments compared to no application. Li et al.^20^ conducted a three-year field experiment on sierozem in Xinjiang, China, which the results indicate that with the increase of N application, SOC storage first increases and then decreases. High N conditions (300 kg ha^–1^) are not conducive to SOC fixation. Zhang et al.^13^ found that first increases and then decreases the net soil C budget (NSCB) with decreasing N fertilizer application rate. The NSCB reached its maximum value when reduced N application of 10% (99kg ha^–1^). Reducing N application of 10% can effectively improve the C sequestration capacity of paddy soil and reduce soil C loss. Peng et al.^21^ reported that N fertilizer input is reduced by 30% based on conventional N application under deep placement of basal fertilizer N and soil fertility of the rice rhizosphere is higher than fertilizer spraying. In addition to directly affecting SOC components, N fertilizer can also affect the accumulation or consumption of SOC by affecting the number and activity of soil microorganisms and the related processes or functions of the C cycle^22–24^. Liu^25^ reported that the agricultural management measures such as covering plastic film and reducing N fertilizer application are beneficial for increasing the proportion of active organic C fractions and improving soil quality. Huang^26^ reported that mineral N significantly impacts the decomposition rate of soil organic C, and a reasonable N application rate balances N input and expenditure, reducing the potential for accumulation and leaching of nitrate N. The specific impact of N fertilizer application on SOC components is not clear^27–29^, which indicates the close relationship between fertilization and the soil C cycle, as well as the complexity of their relationship. The impact of paddy field management on soil carbon pool status and change rate, as well as its ability to improve soil quality, can be characterized by the soil carbon pool management index (CPMI)^30,31^. High levels of soil CPMI indicate high-quality soil conditions. Thus, the CPMI reflects the response of soil C to agronomic management measures such as fertilization and irrigation^32^. Liu et al.^33^ reported that the CPMI can more specifically reflect the characteristics of SOC changes and further demonstrated the rationality of agricultural management measures^34^. Therefore, exploring ways to improve soil C sequestration capacity is essential for improving soil quality and sustainable agricultural development.

The Northeast Black Soil Region is an important grain production and commodity grain export base in China, and the proportion of grain production exceeds 20% of the total national production^26^. This area is the cornerstone of grain production in Northeast China. In addition, protecting the black soil in Northeast China is an important measure to ensure national food security. The black soil in Northeast China has the highest SOC content in arable land. However, due to long-term unreasonable planting and high-intensity utilization, the physical and chemical properties and ecological functions of black soil have deteriorated, and soil fertility has gradually decreased. The decrease in soil organic carbon content in the black soil cultivation layer is the most severe in China, with an average decrease of one-third, and the most severe areas have reached half^25^. Against the backdrop of a sharp decline of black soil productivity and grain security faces serious threats, protecting black soil quality has become particularly important.

To investigate the effects of different N fertilizer management and irrigation regimes on the health of the soil C pool in black soil rice fields under deep placement of basal fertilizer N, a three-year field experiment was conducted to explore the effects of water and N management regimes on soil TOC, EOC, MBC, DOC, CPMI, and rice yield. We hypothesized a significant positive correlation between soil active organic C and rice yield. Reasonable water and N management can improve crop productivity by increasing the soil active organic C content. Our aim was to improve soil C sequestration capacity and enhance soil productivity through reasonable water and N management regimes. This study provides theoretical support for the sustainable development of black soil rice fields in Northeast China.

## Materials and methods

### Overview of study site

**Figure 1:**
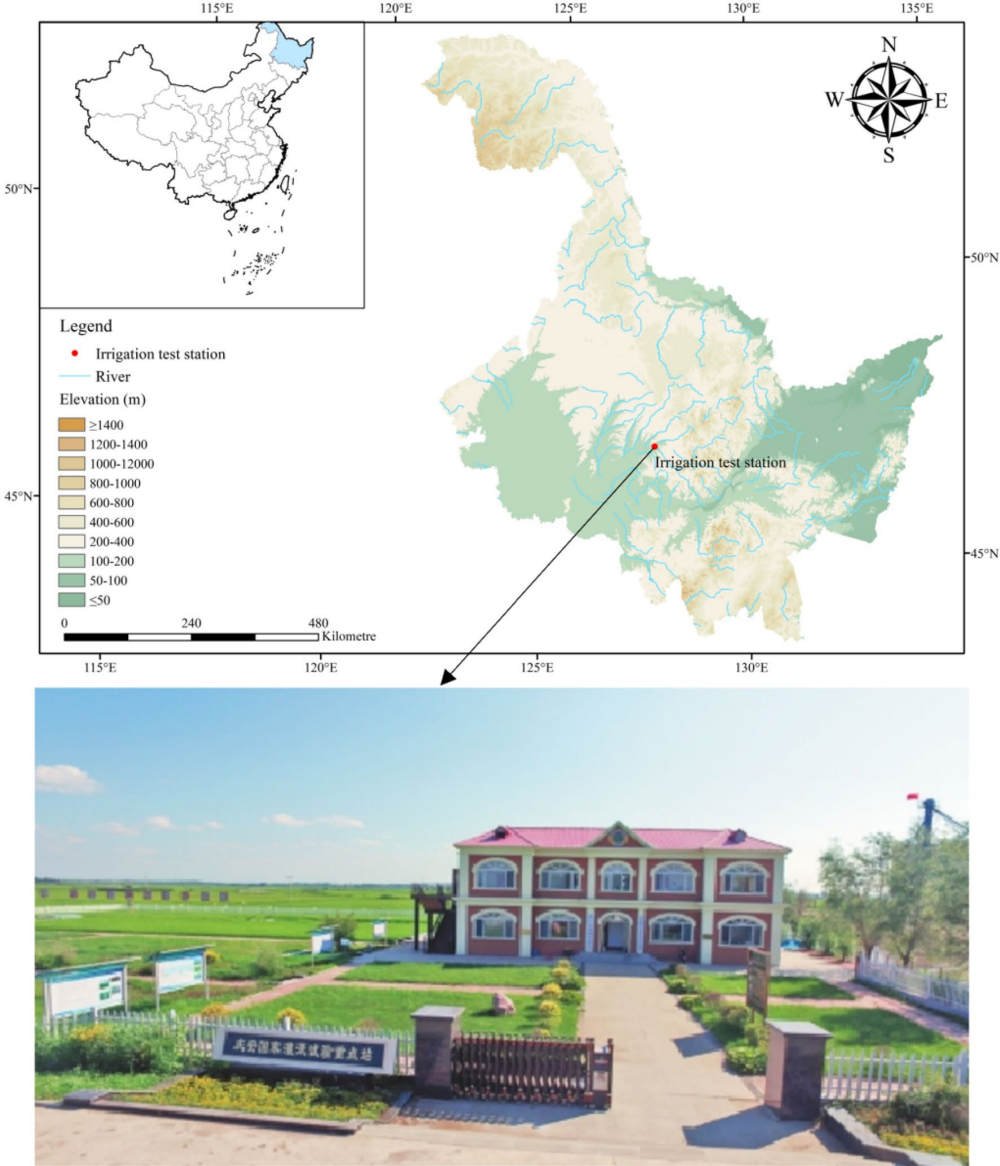
Location of the study area.

This experiment was conducted at the Rice Irrigation Experimental Station (46° 57′ 58″ N, 127° 39′ 39″ E) in Heilongjiang Province, China. The station was in the Heping Irrigation District, Qing’an County, Songnen Plain, Northeast China, as shown in Fig. 1. The soil in the experimental field is the main soil Mollisol type on the Songnen Plain. The soil is fertile, and the nutrient content of the soil is stable. The region has an average annual precipitation of 500–600 mm and a frost-free period of 128 days and has a continental monsoon climate in the cold temperate zone. The major properties of the soil are shown in Table 1.

**Table 1:** The properties of the soil.

Type of soil	Soil layers/cm	Alkaline N/(mg·kg^–1^)	Available P/(mg·kg^–1^)	Available K/(mg·kg^–1^)	pH value	Cation Exchange Capacity mol( +)/(kg)	Particle composition/(%)			Soil texture
							2.0–0.02	> 0.02–0.002	< 0.002	
Mllisols	0–20	185.8	33.5	105.5	6.5	31.4	36.7	31.8	29.5	Sandy clay
	20–40	151.4	29.1	95.6	6.9	25.8	30.5	36.5	29.4	Sandy clay
	40–60	134.6	23.7	89.1	7.1	22.8	27.7	38.9	28.7	Sandy clay

### Experimental design

In this experiment, under deep placement of basal fertilizer N, natural soil (CKT) was used as a blank, and two irrigation regimes were established: conventional flooded irrigation (FI) and controlled irrigation (CI).

The basal fertilizer was applied to the soil at a depth of 4 ~ 5 cm using a side-deeping fertilizer machine (FSPV6; Kubaotian Corporation; Osaka, Japan). The water management practices used for rice are shown in Table 2. A TPIME-PICO64/32 soil moisture tester was used to measure the soil moisture content. When the soil water content was close to or below the lower limit of irrigation, artificial irrigation was applied until the upper limit of irrigation was reached. The N rate (pure N) was set at four levels: Nd0 (0 kg ha^–1^), Nd (110 kg ha^–1^), Nd1 (reduced N application of 10%, 99 kg ha^–1^), and Nd2 (reduced N application of 20%, 88 kg ha^–1^). The experimental design and fertilizer application rates are shown in Table 3. This experiment consisted of 8 treatments, each repeated 3 times. The area of each plot in the experiment was 100 m^2^ (10m × 10m). Each plot was randomly arranged. To prevent the exchange of water and N between each plot, plastic boards with a depth of 40 cm were embedded underground in the ridges of the fields between different experimental plots. The experimental period was from 2018 to 2021, and soil samples after harvest were collected in 2021. The tested rice variety was *Suijing 18*, which is the main locally planted variety. The planting density was 25 holes/m^2^, with 3 plants per hole. Rest of the field management and pest control of rice were the same as those of local farmers.

**Table 2:** Different water management during rice growth stages.

Treatment	Seeding	Early tillering	Field tillering	Later tillering	Jointing and booting	Heading and flowering	Grain-filling	Yellow ripe
CI	0–3 cm	70% *θ*_*s*_	70% *θ*_*s*_	Drainage	80% *θ*_*s*_	80% *θ*_*s*_	70% *θ*_*s*_	Naturally drying
FI	0–3 cm	0–5 cm	0–5 cm	Drainage	0–5 cm	0–5 cm	0–3 cm	Naturally drying

*θ*_*s*_ is the saturated soil moisture content.

**Table 3:** Design of the experimental treatments.

Treatment	Irrigation regimes	Application rate (kg ha^–1^)		
		N	P	K
FNd	Conventional flooded irrigation	110	45	80
FNd1	Conventional flooded irrigation	99	45	80
FNd2	Conventional flooded irrigation	88	45	80
FN0	Conventional flooded irrigation	0	45	80
CNd	Controlled irrigation	110	45	80
CNd1	Controlled irrigation	99	45	80
CNd2	Controlled irrigation	88	45	80
CN0	Controlled irrigation	0	45	80

### Sample handling and data analysis

#### Soil and rice sampling and processing scheme

Undisturbed soil samples were systematically collected using a 100 ml ring knife and a soil drill from both within and outside the treatment plots. Soil samples were collected from the 0–20 cm, 20–40 cm, and 40–60 cm soil layers to capture a comprehensive soil profile. At each depth, three parallel samples were collected to ensure representativeness. Following collection, each soil sample was carefully divided into two portions: One was preserved in its fresh state, while the other was subjected to air drying and subsequent grinding for further analysis (the rice collection in this experiment had obtained permissions, and all procedures were conducted in accordance with the guidelines).

#### Soil sample processing

After drying, soak the soil sample in 15% (v/v) HCl to remove any inorganic C that may exist in the sample, then soak it in deionized water several times to make it pH neutral. After drying again, use a TOC analyzer (Vario EL, Elemental Analysen Systeme GmbH, Germany) to measure the TOC content^35^. The EOC was measured by the 333mM KMnO_4_ oxidation method. We added 333mM KMnO_4_ to the soil sample, which was shaken and centrifuge at 4000 rpm, after which the supernatant was diluted. The absorbance was measured at 565 nm^36^. MBC was measured via the chloroform (CHCl_3_) fumigation method; The soil was divided into two parts, fumigation and unfumigation, and extracted with K_2_SO_4_ solution to determine the organic C content^37^. The soil sample was extracted with 30ml of distilled water for 30 min and shaken, the sample solution was centrifuged and filtered through a 0.45mm nitrocellulose membrane filter. The DOC content of the sample solution was used a TOC analyzer^38^.

#### Calculation of the soil C pool management index

The CPI and LI were calculated using the following equations^39–41^.

1 $$\text{Lability of C }(\text{L})=\frac{\text{EOC}}{{\text{TOC}}-{\text{EOC}}},$$

2 $$\text{Lability Index }({\text{LI}})=\frac{\text{L of each treatment}}{\text{L of CKT}},$$

3 $$\text{Carbon pool index }({\text{CPI}})=\frac{\text{TOC of each treatment}(\text{g}{\text{kg}}^{-1})}{{\text{TO}}\text{C of CKT (g }{\text{kg}}^{-1})},$$

4 $$\text{Carbon pool management index }({\text{CPMI}})= \text{CPI } \times \text{ LI }\times 10.$$

where CPI represents the C pool index, and LI represents the C pool lability index.

#### Statistical analysis

All data calculations were performed with SPSS version 21.0 (Inc., USA), and statistical significance was determined at the 0.05 probability level. Pearson’s correlation was used at p = 0.05 and p = 0.01 for correlation analysis. Origin 2021 was used for plotting and correlation analysis. All tables were visualized using Excel version 2019 (Microsoft, USA).

## Results

### Total organic carbon content (TOC)

**Figure 2:**
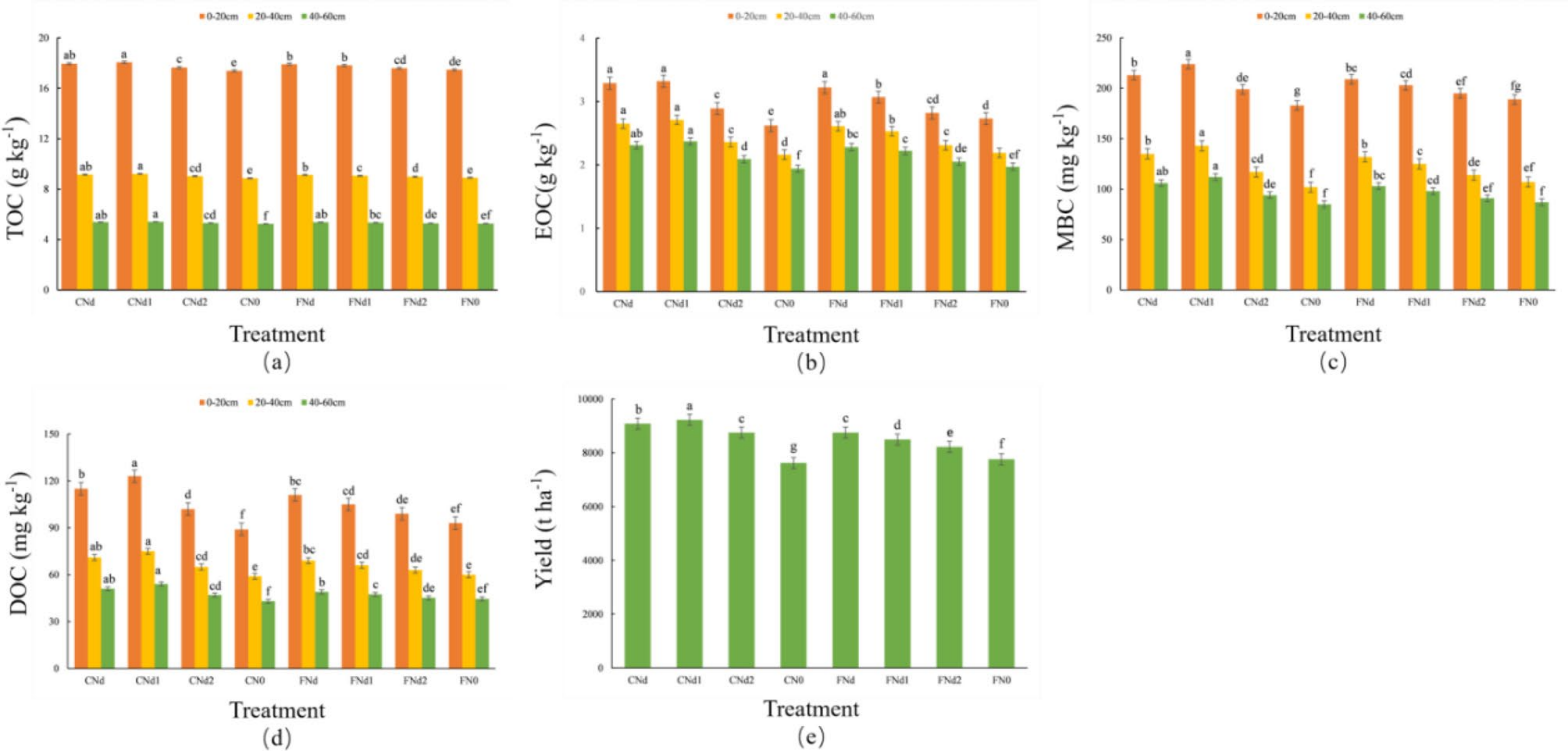
Effects of irrigation and nitrogen management under deep placement of basal fertilizer nitrogen on total organic carbon (**a**), easily oxidizable carbon (**b**), microbial biomass carbon (**c**), dissolved organic carbon (**d**), and yield (**e**). Different lowercase letters above the bars for each site indicate significant differences (P < 0.05) among treatments. Error bars indicate standard deviations.

Figure 2a shows the effect of N fertilizer and irrigation regimes on the soil TOC content. The results of three consecutive years of experiments indicated that the soil TOC content was influenced by N fertilizer management under the two irrigation regimes. The TOC content increased significantly under N fertilizer applied treatments compared to no application (p < 0.05). Under CI, the TOC content did not decrease with decreasing N application rate but instead reached the highest level in the CNd1 treatment. Under FI, the soil TOC content gradually decreased with decreasing N application rate. Compared to those in the CN0 treatment, the TOC content in the CNd, CNd1, and CNd2 treatments increased by 3.22%, 3.85%, and 1.50%, respectively. Compared to those in the FN0 treatment, the TOC content in the FNd, FNd1, and FNd2 treatments increased by 2.52%, 2.00%, and 0.69%, respectively. The irrigation regimes also significantly affected the TOC content in the 0–60 cm soil profile under the different N fertilizer treatments. At the same N fertilizer application rate, the TOC content of CI was greater than that of FI. This increasing trend not only occurred in the 0–20 cm surface soil layer but also in the deep soil layers across the N fertilization treatments. Among them, the TOC content increased by 0.22–1.35% in 0–20 cm soil layer, 0.22%-1.66% in 20–40 cm soil layer, and 0.19%-1.31% in 40–60 cm soil layer. The TOC content gradually decreased with increasing soil depth in each treatment.

### Active carbon fractions

Figure 2b–d shows the effect of N fertilizer and irrigation regimes on the active organic C fractions (EOC, MBC and DOC) contents. The results of three consecutive years of experiments indicated that the active organic C fractions contents were influenced by N fertilizer management under the two irrigation regimes. The active organic C fractions contents increased significantly under N fertilizer applied treatments compared to no application (p < 0.05). Among them, the maximum increase in EOC, MBC, and DOC contents was 26.72%, 40.20%, and 38.20%, respectively. Under CI, the active organic C fractions contents did not decrease with decreasing N application rate but instead reached the highest level in the CNd1 treatment. Under FI, the active organic C fractions contents gradually decreased with decreasing N application rate. The active organic C fractions decreased with the increasing soil depth in every treatment.

The irrigation regimes also significantly affected the active organic C fractions content (p < 0.05). At the same N fertilizer application rate, the active organic C fractions of CI was greater than that of FI. Among them, the maximum increase in EOC, MBC, and DOC contents was 8.14%, 14.40%, and 17.14%, respectively. This increasing trend not only occurred in the 0–20 cm surface soil but was also observed in deep soil layers across the N fertilization treatments. Among them, the EOC and DOC content has the largest growth rate in the 0–20 cm layer, the MBC content has the largest growth rate in the 20–40 cm layer. The CNd1 treatments had higher contents of active organic C fractions at the three soil depths compared to the other treatments. Thus, CI significantly increased the active organic C fractions content, and the addition of an appropriate amount of N fertilizer further enhanced this effect.

### Yield

Figure 2e shows the effect of N fertilizer and irrigation regimes on rice yield. Compared to those in the CN0 treatment, the rice yields in the CNd, CNd1, and CNd2 treatments increased by 19.21%, 21.06%, and 14.84%, respectively. Compared to those in the FN0 treatment, the rice yields in the FNd, FNd1, and FNd2 treatments increased by 12.81%, 9.47%, and 5.97%, respectively. Under CI, the rice yield did not decrease with decreasing N application rate but instead reached the highest level in the CNd1 treatment. This indicated that applying N fertilizer could significantly increase rice yield, but excessive N fertilizer input may not have necessarily led to high yield. The rice yield gradually decreased with decreasing N fertilizer application rate under FI. The irrigation regimes also significantly affected the rice yield. At similar N fertilizer application rate, we observed higher rice yield under CI than FI. Without N fertilizer application, the rice yield was higher under FI than CI. At similar N fertilizer application rate, compared to the FI, the growth rate of rice yield was 3.8% − 8.63% under CI. CI had a more significant effect on increasing rice yield. CNd1 had the maximum rice yield (9.23 t ha^–1^), and CN0 had the minimum yield (7.62 t ha^–1^). Thus, the combination of CI and an appropriate N fertilizer application rate could achieve the higher level of rice yield.

### Carbon pool management index (CPMI)

According to Table 4, the results of field experiments indicated that L, LI, CPI, and CPMI responded similarly to all treatments. N fertilizer and irrigation regimes significantly affected the CPMI. Compared to CKT, the CPMI increased in both the conventional N application rate and reduction 10% N application rate, but decreased in both reduction 20% N application rate and no nitrogen application treatments. This indicated that an excessive reduction N fertilizer application rate was detrimental to the soil C pool health. Under CI, the CPMI first increased and then decreased with decreasing N application rate, and reached the highest level in the CNd1 treatment. The CPMI gradually decreased with decreasing N application rate under FI. At similar N fertilizer application rate (except for CN0 and FN0), the CPMI was higher under CI than FI. Among them, the CPMI increased by 2.57–9.99% in 0–20 cm soil layer, 2.23–10.62% in 20–40 cm soil layer, and 2.16–10.99% in 40–60 cm soil layer. The CPMI is higher under FN0 than CN0.

**Table 4:** Soil carbon pool management index (CPMI) of the three soil layers under the different treatments.

Depth (cm)	Treatments	L	LI	CPI	CPMI
0–20	CKT	0.20 b	1.00 bc	1.00 cd	100.00 d
	CNd	0.22 a	1.11 a	1.01 ab	112.03 ab
	CNd1	0.23 a	1.11 a	1.02 a	113.13 a
	CNd2	0.20 c	0.97 cd	0.99 d	96.11 e
	CN0	0.18 e	0.88 f	0.98 f	85.79 g
	FNd	0.22 a	1.08 a	1.01 abc	109.18 b
	FNd1	0.21 b	1.03 b	1.00 bc	103.15 c
	FNd2	0.19 cd	0.94 de	0.99 de	93.40 e
	FN0	0.19 d	0.91 ef	0.98 ef	89.99 f
20–40	CKT	0.36 bc	1.00 d	1.00 c	100.00 d
	CNd	0.41 a	1.12 a	1.01 a	113.52 ab
	CNd1	0.42 a	1.15 ab	1.02 a	116.85 a
	CNd2	0.35 cd	0.97 e	1.00 c	97.23 de
	CN0	0.32 e	0.89 f	0.98 e	86.89 f
	FNd	0.40 a	1.10 b	1.01 ab	111.22 b
	FNd1	0.39 ab	1.07 c	1.00 bc	106.82 c
	FNd2	0.35 cde	0.95 e	0.99 cd	94.61 e
	FN0	0.33 de	0.90 f	0.99 de	88.37 f
40–60	CKT	0.66 d	1.00 d	1.00 cd	100.00 d
	CNd	0.75 ab	1.14 b	1.01 ab	114.86 b
	CNd1	0.78 a	1.18 a	1.02 a	119.67 a
	CNd2	0.65 de	0.98 d	1.00 de	97.79 de
	CN0	0.59 f	0.89 f	0.98 f	87.40 f
	FNd	0.74 bc	1.11 b	1.01 abc	112.42 b
	FNd1	0.71 c	1.07 c	1.00 bcd	107.81 c
	FNd2	0.63 e	0.96 e	0.99 def	94.97 e
	FN0	0.60 f	0.90 f	0.99 ef	89.36 f

Different letters indicate significant differences (P < 0.05) among different treatments

### Relationships among TOC, active C fractions, CPMI and yield

**Figure 3:**
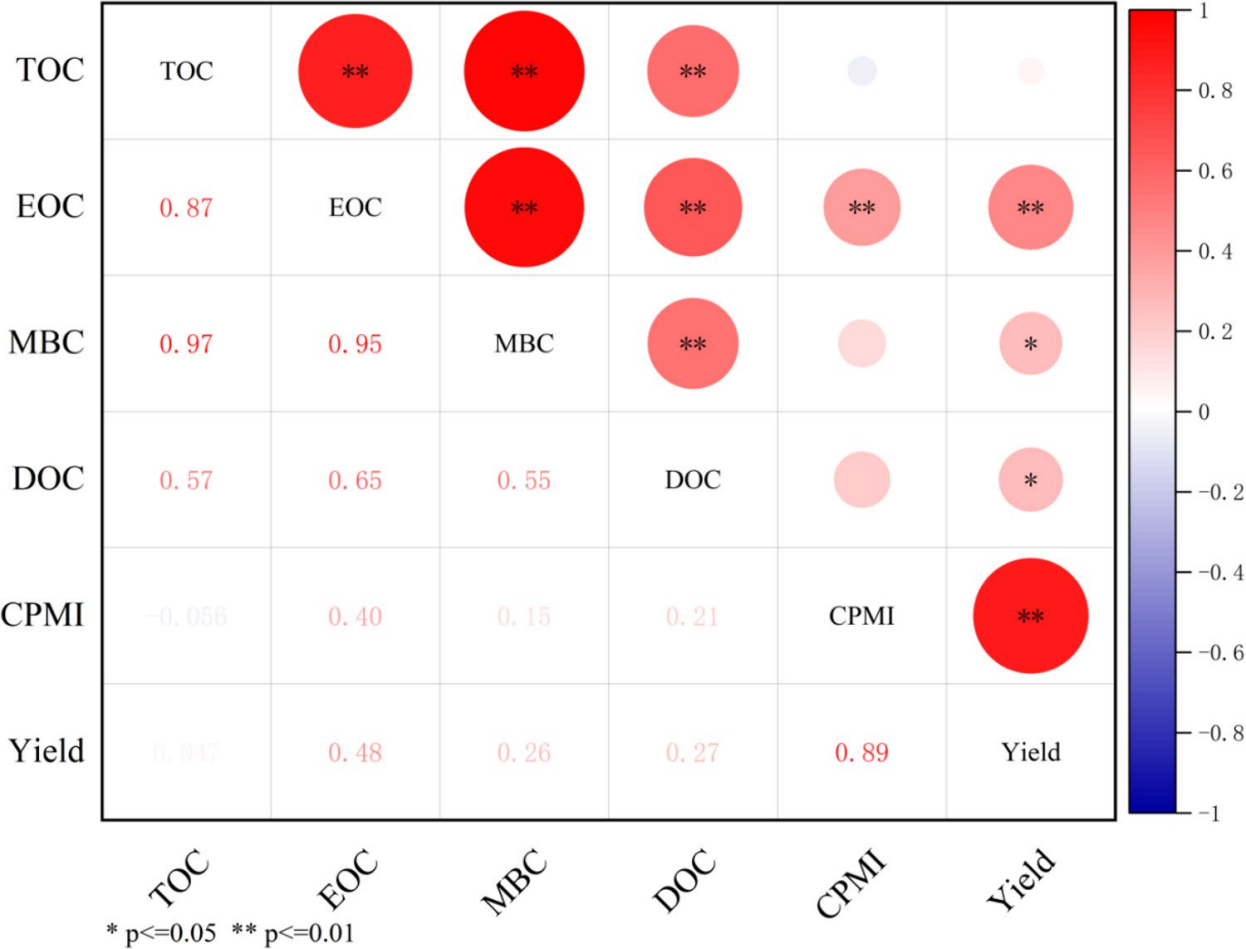
Pearson correlations between TOC, active C fractions, CPMI and yield.

According to Fig. 3, the soil TOC was significantly related to MBC, EOC, and DOC at the 0–60 cm soil depth. There were significant correlations between the active organic C fractions. The CPMI was significantly related to soil EOC. The soil DOC, MBC, EOC, CPMI and rice yield were significantly positively correlated. Thus, fertilization and irrigation regimes in the field not only affected the TOC content of the soil but also changed the CPMI by affecting active organic C fractions in the soil.

## Discussion

### Effect of different water and N management practices on the TOC content

In our study, the variations in total organic carbon (TOC) content were relatively smaller compared to soil organic carbon (SOC), mainly due to their sensitivity to management changes^42^. Compared to treatments without nitrogen (N) fertilizer application, the TOC content increased with deep placement of basal fertilizer N. This is attributed to the promotion of crop root growth and increased input of root stubble and exudates into the soil, thereby enhancing TOC content^43^. These findings align with those of Xiao et al.^44^ and Chen et al.^45^. Conversely, Ruan et al.^46^ reported that N fertilizer application reduces SOC content, highlighting the influence of irrigation regimes, climate, soil type, and tillage methods on SOC response.

At similar N fertilizer application rate, we observed higher TOC content under CI than FI. This may be due to the improvement in soil aeration through CI. The oxidative activity of roots can delay root senescence and increase nutrient absorption during the aerobic period^47^. In addition, the decrease in water supply in rice fields negatively affects the leaf area index, leading to adverse effects on aboveground biomass accumulation, accelerating rice senescence, increasing litter input into the soil^48,49^, or promoting root deposition^50^. These processes contribute to increased TOC.

The highest TOC content was observed in the CNd1 treatment group, indicating that the combination of CI and an appropriate N fertilizer application rate strengthened TOC enrichment. Furthermore, TOC decreased with increasing soil depth across treatments, suggesting that N fertilizer application rate and irrigation regime influence the vertical distribution of TOC. Similar conclusions have been drawn by other researchers^51,52^.

### Effect of different water and N management practices on active C fractions

Numerous studies have shown that N fertilizer application rates and irrigation regimes can alter the active organic C fractions of soil in the short term^53,54^ due to their rapid response and high sensitivity to management practices and environmental changes. Compared to treatments without N fertilizer application, the DOC, EOC, and MBC content significantly increased at the three soil layers under both irrigation regimes. This may be due to the N fertilizer application being beneficial in providing a large amount of substrate for soil urease catalysis, thereby enhancing urease activity. Furthermore, applying N fertilizer being beneficial in improving the N nutrition of soil microorganisms and promoted their reproduction^44^, which contribute to increased the soil active organic C contents.

At similar N fertilizer application rate, we observed higher active organic C fractions content under CI than FI. This may be due to the improvement in root growth through CI, which considered a C source for soil microbial activity^55^. In addition, CI is beneficial in improving soil catalase activity and promoting the decomposition of hydrogen peroxide into water and oxygen than FI. Hydrogen peroxide reduced its toxic effect on soil and created a suitable field environment for microbial activity^56^, promoting the binding of soil particles and macroaggregates, thereby enhancing active organic C content. Under CI, Our research reported that the active organic C fractions contents did not decrease with decreasing N application rate but instead reached the highest level in the CNd1 treatment. This may be due to the physical and chemical properties of the paddy field. High N input increases soil acidity and inhibits microbial activity, resulting in higher active organic C content under CNd1 than CNd^57^.

### Effect of different water and N management practices on rice yield

The dynamic balance of the soil C pool is closely related to crop nutrition and soil management, which directly affects crop yield and soil fertility^58^. Therefore, well-managed soil can support sustainable production and improve crop yields. Our study showed that the interaction between irrigation and N fertility affects rice yield. At similar N fertilizer application rate, we observed higher rice yield under CI than FI. This result was most likely due to the promoting effect of N mineralization and effectiveness is greater than the inhibitory effect caused by water deficiency under CI. CI had a significant positive impact on rice yield. This finding was consistent with the observed changes in the organic C pool under CI. There was a significant positive correlation between soil MBC, EOC, and DOC and rice yield, indicating that CI can improve crop productivity by enhancing the soil active organic C content. Similar conclusions have been drawn by Qin et al.^59^. The highest rice yield was observed in the CNd1 treatment group, indicating that the combination of CI and an appropriate N fertilizer application rate has a more significant effect on increasing rice yield.

### Effect of different water and N management practices on the CPMI

The CPMI is an effective indicator for evaluating changes in soil quality under different measures^36^. Our research showed that different irrigation regimes and N fertilizer applications regulated the CPMI. This indicated that soil has a large capacity for supplying nutrients for crop growth^60^. The changes in CPMI and soil active C fractions were similar and significantly correlated with EOC. Similar conclusions have been drawn by Yuan et al.^34^. There was a significant positive correlation between soil EOC, MBC, DOC, and TOC in the 0–60 cm soil layer, indicating that TOC is a key factor affecting the active organic C fraction. The changes in soil active C fractions can effectively indicate the direction of TOC changes^61^. Our research reported that the combination of CI and an appropriate N fertilizer application rate can improve soil quality and agricultural productivity. In addition, MBC, DOC, and CPMI may be a sensitive indicator of the impact of different N fertilizer application rates and irrigation regimes on soil fertility. Future study will focus on how soil factors, active C pools and soil microorganisms pools change under CI and N application. The water-nitrogen interaction effect provides an opportunity for the sustainable development of low water and N reduction in rice production.

## Conclusion

In this study, after 3 years, compared to treatments without nitrogen (N) fertilizer application, the TOC content, active SOC fractions content, rice yield increased with deep placement of basal fertilizer N. At similar N fertilizer application rate, we observed higher rice yield and TOC, EOC, MBC, and DOC content under CI than FI. This increasing trend not only occurred in the 0–20 cm surface soil layer but also in the deep soil layers across the N fertilization treatments. Compared to that of FI, the rice yield growth rate of CI was 3.8–8.63%. Compared to those in the CKT treatment, the CPMI of both the conventional N application rate and the 10% N application rate reduction treatments increased under the two irrigation regimes, while that of the other treatments decreased. Fertilization and irrigation regimes in the field not only affected the TOC content of the soil but also changed the CPMI by affecting active organic C fractions in the soil. The highest rice yield, TOC content, active C fractions, and CPMI was observed in the CNd1 treatment group. Our results showed low expectations of the effect of too much irrigation and a high N fertilizer application rate. From the perspective of enhancing soil C fixation and increasing rice yield, the optimal N application rate for CI under deep placement of basal fertilizer N was 99 kg ha^–1^, and the greatest effect was achieved by adjusting water with fertilizer and promoting fertilizer with water.

## Data Availability

The datasets generated and/or analysed during the current study are available from the corresponding author on reasonable request.
